# Allelic variation within the *S*-adenosyl-L-homocysteine hydrolase gene family is associated with wood properties in Chinese white poplar (*Populus tomentosa*)

**DOI:** 10.1186/1471-2156-15-S1-S4

**Published:** 2014-06-20

**Authors:** Qingzhang Du, Lu Wang, Daling Zhou, Haijiao Yang, Chenrui Gong, Wei Pan, Deqiang Zhang

**Affiliations:** 1 National Engineering Laboratory for Tree Breeding, Beijing Forestry University, Beijing 100083, P. R. China; 2 Key Laboratory of Genetics and Breeding in Forest Trees and Ornamental Plants, Ministry of Education, Beijing Forestry University, Beijing 100083, P. R. China

**Keywords:** Haplotype-based association test, Linkage disequilibrium (LD)-linkage analysis, *Populus tomentosa*, S-Adenosyl-l-homocysteine hydrolase

## Abstract

**Background:**

S-adenosyl-l-homocysteine hydrolase (SAHH) is the only eukaryotic enzyme capable of S-adenosyl-l-homocysteine (SAH) catabolism for the maintenance of cellular transmethylation potential. Recently, biochemical and genetic studies in herbaceous species have obtained important discoveries in the function of *SAHH*, and an extensive characterization of *SAHH *family in even one tree species is essential, but currently lacking.

**Results:**

Here, we first identified the SAHH family from *Populus tomentosa *using molecular cloning method. Phylogenetic analyses of 28 SAHH proteins from dicotyledons, monocotyledons, and lower plants revealed that the sequences formed two monophyletic groups: the PtrSAHHA with PtoSAHHA and PtrSAHHB with PtoSAHHB. Examination of tissue-specific expression profiles of the *PtoSAHH *family revealed similar expression patterns; high levels of expression in xylem were found. Nucleotide diversity and linkage disequilibrium (LD) in the *PtoSAHH *family, sampled from *P. tomentosa *natural distribution, revealed that *PtoSAHH *harbors high single-nucleotide polymorphism (SNP) diversity (π=0.01059±0.00122 and 0.00930±0.00079,respectively) and low LD (r^2 ^> 0.1, within 800 bp and 2,200 bp, respectively). Using an LD-linkage analysis approach, two noncoding SNPs (*PtoSAHHB_1065* and *PtoSAHHA_2203*) and the corresponding haplotypes were found to significantly associate with α-cellulose content, and a nonsynonymous SNP (*PtoSAHHB_410*) within the SAHH signature motifs showed significant association with fiber length, with an average of 3.14% of the phenotypic variance explained.

**Conclusions:**

The present study demonstrates that *PtoSAHHs *were split off prior to the divergence of interspecies in *Populus*, and *SAHHs *may play a key role promoting transmethylation reactions in the secondary cell walls biosynthesis in trees. Hence, our findings provide insights into SAHH function and evolution in woody species and also offer a theoretical basis for marker-aided selection breeding to improve the wood quality of *Populus*.

## Background

In plants, animals, and microorganisms, transmethylation reactions are commonly involved in modifications of almost all metabolites. In most methylation reactions, *S*-adenosylmethionine (SAM) is the methyl group donor used by all organisms, and *S*-adenosyl-L-homocysteine (SAH) is formed as a by-product of the reaction after the methyl group donor is transferred to acceptors [1,2]. SAH is a strong product inhibitor of SAM-dependent methyltransferases and is hydrolyzed by *S*-adenosyl-L-homocysteine hydrolase (SAHH) to homocysteine and adenosine, which is the only eukaryotic enzyme capable of SAH catabolism. In addition, the enzymatic activity of SAHH is related to the ratio of SAM to SAH; the accumulation of SAH inhibits SAHH activity, thereby reducing both the methylation status and gene expression.

SAHH was first described as a single enzymatic entity by de la Haba and Cantoni [3], although researchers have known since 1955 that SAH undergoes enzymatic breakdown when incubated with crude rat liver extracts [4]. In the same year, SAH was chemically characterized as the product derived from SAM via transmethylation [5], a reaction first revealed by the pioneering studies of Cantoni and Scarano [6]. To date, full-length *SAHH *has been isolated from many microorganisms, including the archaeon *Sulfolobus solfataricus *[7], *Saccharomyces cerevisiae, Trypanosoma cruzi*, and *Chlamydomonas *sp. ICE-L [8,9]. In addition, *GhSAHH *from *Gossypium hirsutum, CsSAHH *from *Cucumis sativus*, and *SAHH *from *Volvariella volvacea *have been cloned in plants [10,11]. Several mutants created by an SAHH deficiency have been characterized from various plant species. For example, tobacco plants expressing an *SAHH *antisense transgene exhibit abnormal floral organs, stunted growth, and delayed senescence [12]. A point mutation in the *Arabidopsis SAHH1 *was expressed abnormally with slow growth, low fertility, and poor germination [13]. Antisense expression of *SAHH *in petunia is associated with delayed flowering, increased leaf size, and higher seed yield [14]. Although biochemical and genetic studies in herbaceous species have obtained important discoveries in understanding the function of *SAHH*, the functions of other *SAHH *family members in even one tree species remain unknown.

In trees, a marker-assisted selection (MAS) strategy is essential to dissect complex traits into their genetic components to further improve conventional tree breeding [15,16]. Linkage disequilibrium (LD)-based association studies, also known as LD mapping, are an effective approach of providing an understanding between complex quantitative traits and underlying genetic variation in natural or breeding populations [17]. Previous studies have demonstrated that LD mapping can be used to identify allelic variations associated with quantitative traits, such as those pertaining to wood property, disease resistance, and drought tolerance [18-20], suggesting that the new approach plays a particularly useful role in forest tree breeding programs. For example, 27 significant single-marker associations across 40 candidate genes in three composite traits were found in black cottonwood [21]. In addition, a recent study showed that nine significant single-nucleotide polymorphism (SNP) associations from six genes with diverse roles in cambial development associated with wood or growth traits were identified in a discovery population of *Corymbia citriodora *subsp. *variegata *[22].

In the present study, *Populus *was used as a model to address the structure, function, and evolution of the *SAHH *gene family in trees. Using molecular cloning method, we first identified two *SAHH *family members (*PtoSAHHA *and *PtoSAHHB*) from a cDNA library of mature xylem from *Populus tomentosa*. Real-time polymerase chain reaction (PCR) revealed that the high transcript abundance in developing and mature xylem may indicate their important role in secondary cell wall formation. Subsequently, we detected nucleotide diversity and LD decay within this gene family. SNP- and haplotype-based association tests were then used to examine allelic variation with putative function on growth and wood-property traits in both association (discovery) population and linkage (validation) population studies on *P. tomentosa*. The comprehensive study of *PtoSAHH *family members improves our understanding of the regulatory mechanism of the gene family in secondary cell wall formation.

## Results

### Isolation and sequence analysis of *PtoSAHH *family members

Two full-length cDNAs from *PtoSAHHA *and *PtoSAHHB *were isolated from a cDNA library prepared from the mature xylem zone of *P. tomentosa *using reverse transcription (RT)-PCR amplification. Two complete sequences were deposited in GenBank under Accession Nos. KF467170 and KJ198848, and consisted of the 5' terminal untranslated region (UTR) of 229 bp and 129 bp, the 3'-UTR of 248 bp and 181 bp, and coding regions of 1,968 bp and 2,131 bp, respectively. An equal open reading frame (ORF) of 1,458 bp was found that encoded a polypeptide of 485 amino acids in both *PtoSAHHA *and *PtoSAHHB *(Table 1). These two *PtoSAHH *cDNAs shared 88.8% nucleotide sequence identity, and were 81.7% and 80.7% identical, respectively, to *AtSAHH *(AY150471.1). The predicted molecular weight of PtoSAHHA and PtoSAHHB were 53.17 kDa and 53.36 kDa (Table 1), respectively, which were approximately equivalent to proteins of SAHH in other plants. PtoSAHHA and PtoSAHHB showed high similarity (90.1-98.1%) with SAHHs from *P. trichocarpa, Arabidopsis*, cotton, rice, and maize.

**Table 1:** Identification of *PtoSAHHA *and *PtoSAHHB *in *Populus*

Gene	cDNA (GenBank)	Genomics (GenBank)	Genomic DNA length (bp)	cDNA length (bp)	Protein size		p*I*
						
					Amino acids	kDa	
*PtoSAHHA*	KF467170	KF467171	2,445	1,935	485	53.17	5.79
*PtoSAHHB*	KJ198848	KJ198849	2,441	1,768	485	53.36	6.15

**Figure 1:**
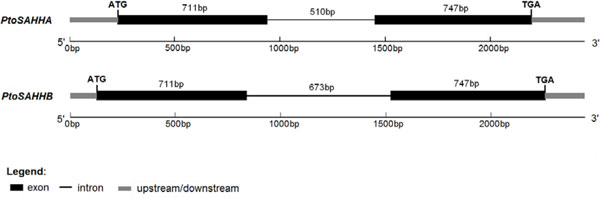
**Exon-intron structure of *PtoSAHHA *and *PtoSAHHB***. Exons are shown in black boxes. Lines between boxes indicate introns. Gray boxes represent upstream and downstream.

Next, a genomic scale search revealed gene structures of *PtoSAHHA *and *PtoSAHHB *(GenBank Accession Nos. KF467171 and KJ198849), as shown in Figure 1. The two full-length genomic sequences (2,445 bp and 2,441 bp) consisted of two exons (711 bp and 747 bp in both *PtoSAHHA *and *PtoSAHHB*) separated by one intron (510 bp in *PtoSAHHA *and 673 bp in *PtoSAHHB*). Introns started with a 5' G-T and ended with a 3' A-G, which were in accordance with the GT-AG rule for a splice site. The two genomic DNAs shared high sequence similarity at the nucleotide level (80.4%).

### Proteomic and phylogenetic analyses of PtoSAHHs

**Figure 2:**
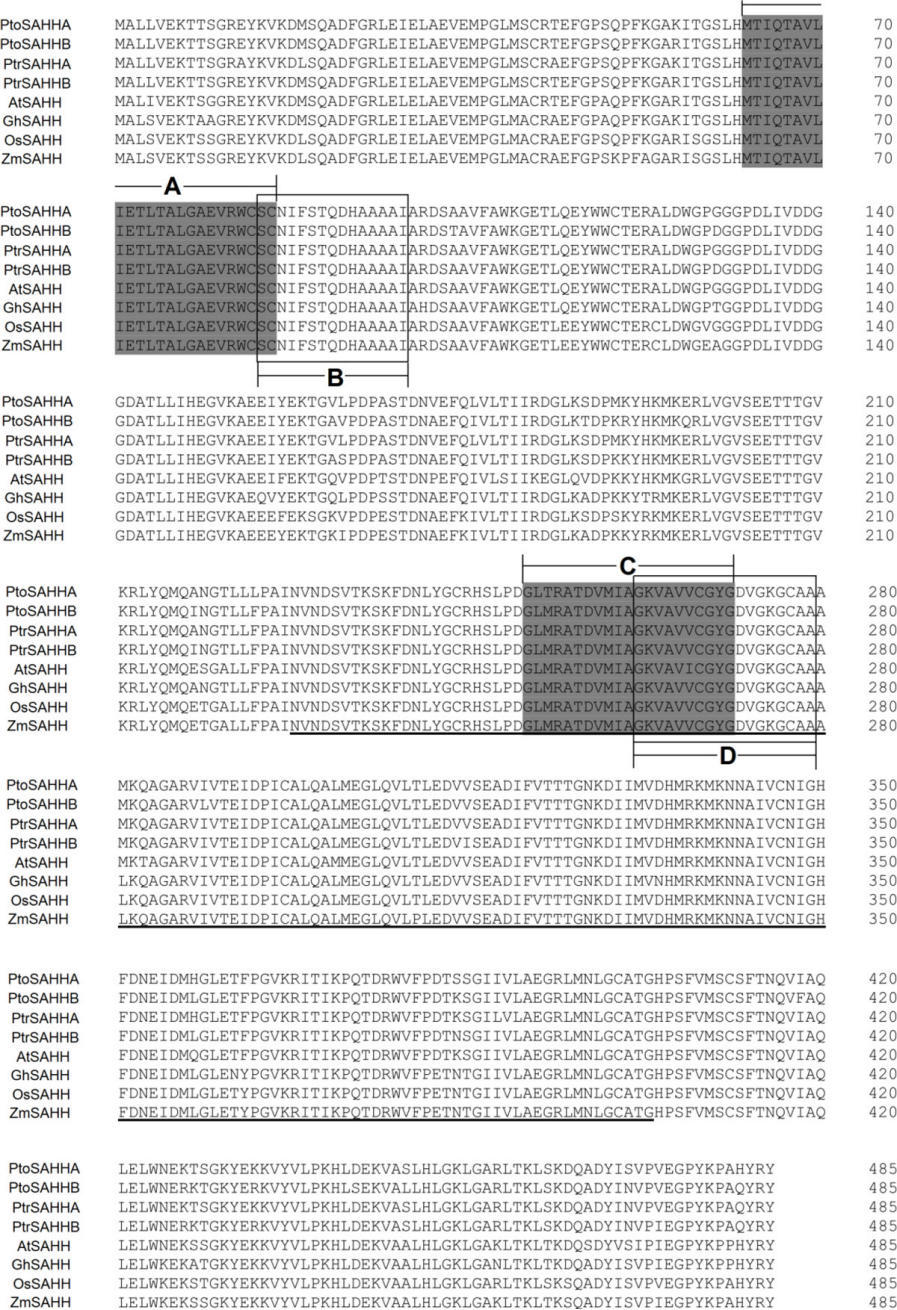
**Protein sequence alignment of PtoSAHHA and PtoSAHHB with other plant PtoSAHHs**. Numbers on the right represent positions of amino acids in each protein. Transmembrane domains (A and C) are shaded. SAHH signature motifs (B and D) are indicated in boxes. NAD-binding domain is underlined. Detailed information on these genes is presented in Table 5.

Blast analysis indicated that the deduced amino acid sequences of *PtoSAHHA *and *PtoSASHHB *shared high homology with the SAHH of other model plants (Figure 2), suggesting they should be members of this protein family. Like any other SAHHs of *P. trichocarpa, Arabidopsis*, cotton, rice, and maize, both PtoSAHHs contained one characteristic AdoHcyase NAD-binding domain and two transmembrane domains at residues 63-86 and 251-271 (Figure 2). Using ExPASY-PROSITE software (http://www.expasy.org/prosite/), two SAHH signature motifs were predicted near the transmembrane domains at residues 85-99 and 262-279 (Figure 2).

**Figure 3:**
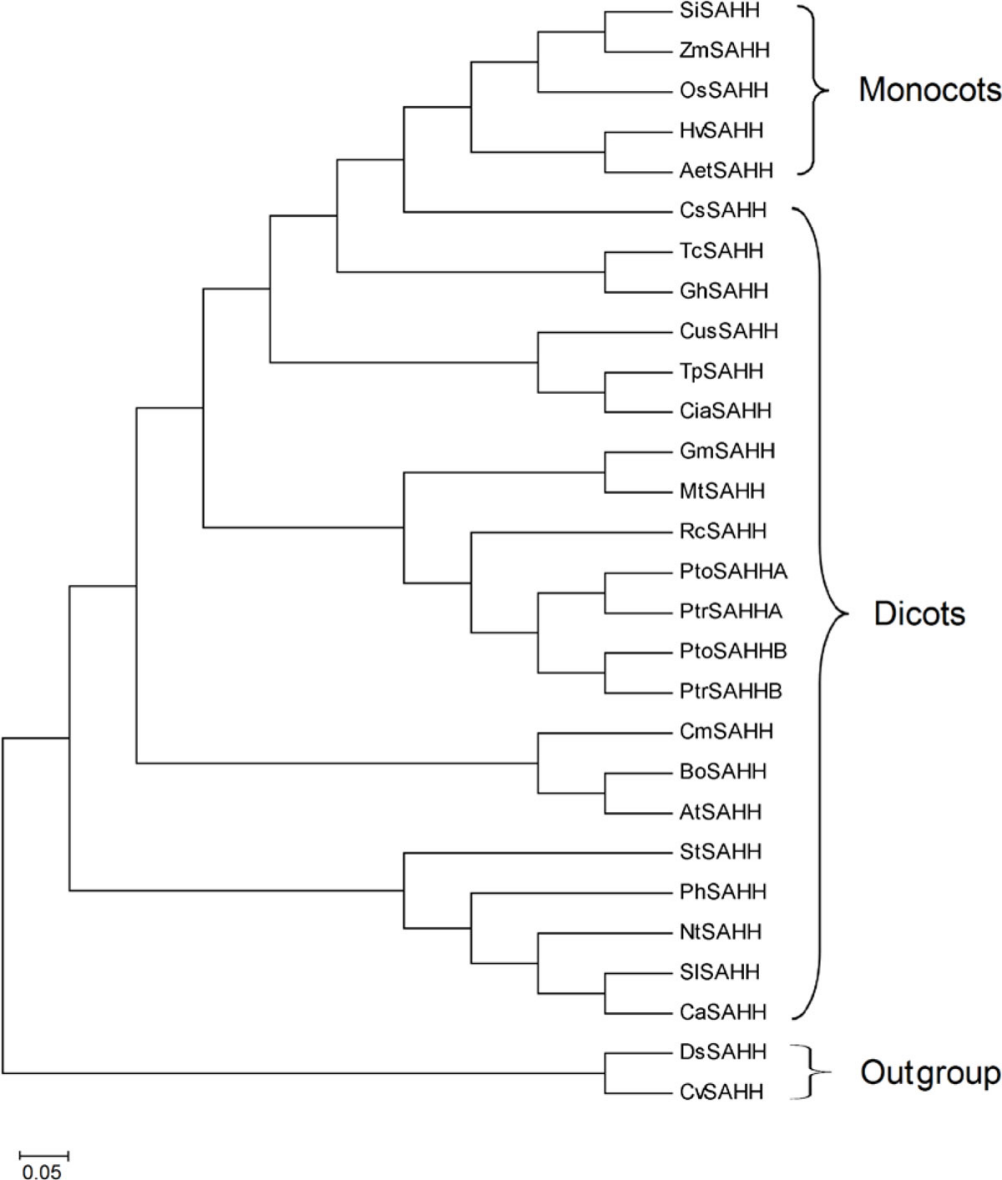
**Neighbor-joining phylogenetic tree of SAHH family members**. Detailed information on all protein species is presented in Table 5.

To analyze the evolutionary relationship between poplar SAHH proteins and SAHHs from other plants, a rooted neighbor-joining (NJ) tree was constructed using a multiple sequence alignment of poplar SAHH proteins and sequences from additional plants, including dicotyledons (*P. trichocarpa *and *A. thaliana*) and monocotyledons (*Oryza sativa *and *Zea mays*), as well as lower plants, such as *Chlorella variabilis *and *Dunaliella salina *(Table S1 in Additional file 1). As shown in Figure 3, 28 SAHH sequences formed two monophyletic groups, terrestrial and aquatic plants, with well-supported bootstrap values. Further subdivisions showed that the terrestrial groups could be classified into monocotyledons and dicotyledons (Figure 3), suggesting that SAHHs split off before the divergence of monocots and dicots ~200 million years ago [23]. The pattern of PtrSAHHA/PtoSAHHA and PtrSAHHB/PtoSAHHB suggests that the *SAHH*s were split off prior to the divergence of interspecies in *Populus*.

### Transcript profiling of mRNAs for *PtoSAHHs *in tissues and organs

**Figure 4:**
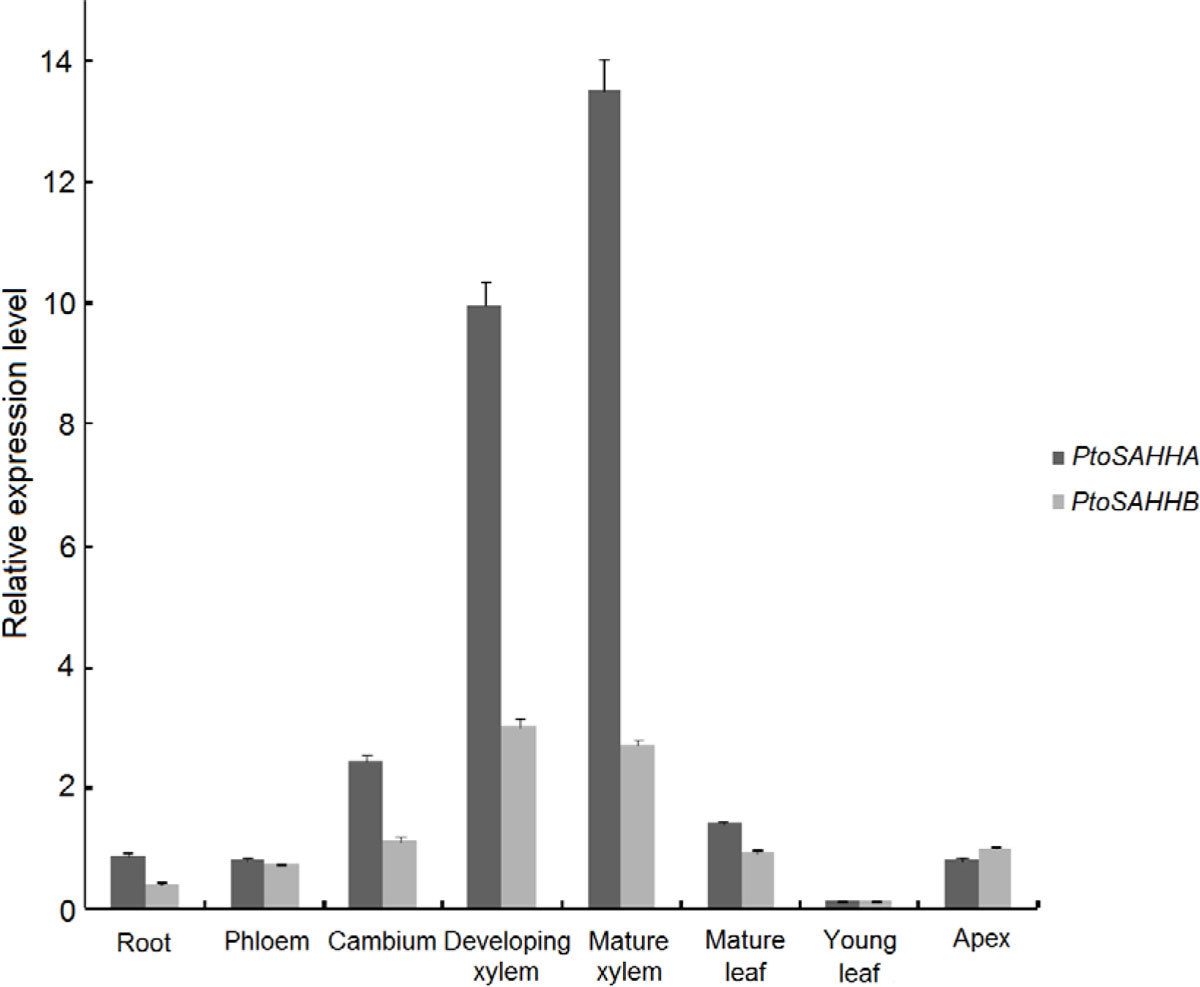
**Relative transcript abundance of *PtoSAHHA *and *PtoSAHHB *in tissues and organs**. Expression levels were normalized to the mean value of *actin*.

Transcript accumulation of *PtoSAHHA *and *PtoSAHHB *was profiled by real-time quantitative RT-PCR to compare steady mRNA levels in various organs and tissues of *P. tomentosa *(Figure 4) with gene-specific primers (Table S2 in Additional file 1). Transcript abundances of the two genes accumulated preferentially in the developing xylem and mature xylem, and gave similar profiles overall (Figure 4). *PtoSAHHA *transcript levels were highest in mature xylem (13.51) and developing xylem (9.97), and also high in cambium (2.441) and mature leaf (1.403). Compared with *PtoSAHHA, PtoSAHHB *showed less transcript accumulation profiles across all organs and tissues examined. The transcripts of *PtoSAHHB *were predominantly detectable in developing xylem (3.031) and mature xylem (2.696). Medium levels of expression were found in cambium (1.132), apex (0.9879), and the mature leaf (0.9395). In the young leaf, both *PtoSAHHA *and *PtoSAHHB *showed the lowest expression levels (0.1143 and 0.1345). Given the results described above, the higher expression levels of the two genes in the developing xylem imply that *PtoSAHHA *and *PtoSAHHB *may significantly contribute to cell wall thickening in wood.

### Nucleotide diversity and linkage disequilibrium of *PtoSAHHs *in discovery populations

Genomic sequences of *PtoSAHHA *and *PtoSAHHB*, ~2,445 bp and ~2,441 bp in length, respectively, were isolated from 43 unrelated individuals encompassing nearly the entire natural range of *P. tomentosa*. All 86 sequences from *PtoSAHHA *and *PtoSAHHB *identified in 43 unrelated individuals were deposited in GenBank databases (KF467172-KF467214 and KJ198849-KJ198891). Statistical analysis of nucleotide variation (excluding indels) over various regions of *PtoSAHHA *and *PtoSAHHB *are summarized in Table 2. In total, 326 SNPs were found in the 4,886 bp sequenced from the two genes (166 from *PtoSAHHA *and 160 from *PtoSAHHB*), or one SNP every 15 bp. The distribution of SNP frequencies in various gene regions are as follows: 13 bp^-1 ^in the 5'-UTR, 16 bp^-1 ^in exons, 12 bp^-1 ^in introns, and 16 bp^-1 ^in the 3'-UTR of *PtoSAHHA*; 14 bp^-1 ^in the 5'-UTR, 18 bp^-1 ^in exons, 11 bp^-1 ^in introns, and 14 bp^-1 ^in the 3'-UTR of *PtoSAHHB*. The two genes displayed a lower SNP density in coding regions compared to noncoding regions, suggesting that the coding region is conserved relative to other regions under natural pressure.

**Table 2:** Summary of nucleotide polymorphisms within *PtoSAHHA and PtoSAHHB*

Locus	Region	**Length **(bp)	No. of polymorphic sites	Frequency (bp^-1^)	Nucleotide diversity	
					
					π_T_	θ_w_
*PtoSAHHA*	5'-UTR	229	18	13	0.01766	0.01798
	Exon 1	711	45	16	0.00710	0.01447
	Synonymous	163.01	17	10	0.02308	0.02385
	Nonsynonymous	547.99	28	20	0.00235	0.01169
	Intron 1	510	43	12	0.01401	0.02033
	Exon 2	747	44	17	0.00758	0.01378
	Synonymous	172.01	19	9	0.02524	0.02503
	Nonsynonymous	571.99	25	23	0.00231	0.01056
	3'-UTR	248	16	16	0.01640	0.01493
	Total silent^a^	1307.03	113	12	0.01767	0.02001
	Synonymous	335.03	36	9	0.02418	0.02498
	Nonsynonymous	1,119.97	53	21	0.00233	0.01104
	Total^b^	2,445	166	15	0.01059	0.01574

*PtoSAHHB*	5'-UTR	129	9	14	0.01094	0.01612
	Exon 1	711	44	16	0.00919	0.01430
	Synonymous	163.29	17	10	0.02590	0.02406
	Nonsynonymous	547.71	27	20	0.00421	0.01139
	Intron 1	673	59	11	0.01772	0.02051
	Exon 2	747	35	21	0.00248	0.01083
	Synonymous	174.08	9	19	0.00291	0.01195
	Nonsynonymous	569.92	26	22	0.00236	0.01054
	3'-UTR	181	13	14	0.00568	0.01707
	Total silent^a^	1310.38	107	12	0.01444	0.01887
	Synonymous	337.38	26	13	0.01404	0.01781
	Nonsynonymous	1,117.62	53	21	0.00326	0.01096
	Total^b^	2,441	160	15	0.00930	0.01523

Regions containing indels were excluded from the calculation; the standard deviations (SD) of π_T _was not shown in this table; ^a^Total silent = synonymous plus noncoding sites; ^b^Total = silent sites plus nonsynonymous sites.

Nucleotide diversity was calculated using the average number of nucleotide differences per site between two sequences (π) and the population mutation parameter (θ) for each gene separately per region, as well as overall. In general, both *PtoSAHHA *and *PtoSAHHB *showed high nucleotide diversity with π=0.01059±0.00122 and 0.00930±0.00079, and θ=0.01574±0.00312 and 0.01523±0.00288, respectively (Table 2). Nucleotide diversity of different gene regions varied significantly in that π ranged from 0.00710±0.00092 (exon 1) to 0.01766±0.00172 (5'-UTR) in *PtoSAHHA*, and from 0.00248±0.00067 (exon 2) to 0.01772±0.00135 (intron 1) in *PtoSAHHB *(Table 2). Based on all homologous DNA sequences data from different species (Table S1 in Additional file 1), within coding regions of *SAHHAs *and *SAHHBs*, the average of nonsynonymous nucleotide diversity $\left(d_{N},\pi =0.00483\pm 0.00032\text{ and }0.00988,\pm 0.00065,\text{respectively}\right)$ was 7.3- and 2.5-fold smaller than synonymous nucleotide diversity $\left(d_{S},\pi =0.03510\pm 0.00120\;\text{and }0.02526\pm 0.00151,\text{respectively}\right)$. The *d_N_/d_S _*values for exons were < 1, indicating strong purifying selection is involved in evolving *SAHHs *during species speciation. Of all the SNPs in *PtoSAHHA *and *PtoSAHHB*, 222 were singletons and 104 were common sites (frequency ≥ 0.05; Table 3). Further analysis revealed that 255 of 326 were transitions (78.2%) and 71 of 326 were transversions (21.8%); the ratio of transitions to transversions was 3.59:1 (Table 3).

**Table 3:** Summary of transitions and transversions for SNPs identified in the *PtoSAHH *family

Name	No. of total SNPs	No. of common SNPs*	No. of singleton SNPs	Transitions		Transversions				Transitions: transversions
						
				A = G	T = C	A = C	G = T	A = T	G = C	
*PtoSAHHA*	166	53	113	47	88	2	10	11	8	4.35
*PtoSAHHB*	160	51	109	48	72	6	9	16	9	3.00
*Total*	326	104	222	95	160	8	19	27	17	3.59

*Common SNPs representing the minor allelic frequency is ≥ 5%.

Using nucleotide diversity data from both *PtoSAHHA *and *PtoSAHHB*, the results from within- or among-climatic region differentiation suggested similar patterns among π_T_, π_sil_, π_syn_, and π_nonsyn _(Table 4), indicating that the level of selective constraint was similar among climatic regions. Tajima's *D *[24] and Fu and Li's *D *[25] statistics were used to determine whether a gene or genomic region was evolving randomly (neutral evolution) or under selection (non-neutral evolution). No significant departures from the neutral evolution were identified using Tajima's *D *among all three climatic regions and the whole *P. tomentosa *population in both *PtoSAHHA *and *PtoSAHHB *(Table 4). Fu and Li's *D *statistical tests were negative for all three regions and the whole population in both genes, with significant departure observed in the whole population (*P *< 0.05; Table 4), revealing an excess of low-frequency polymorphisms in the species-wide samples. Indeed, 113 of 166 variants in *PtoSAHHA *and 109 of 160 variants in *PtoSAHHB *were singletons, accounting for 68.07% and 68.13%, respectively, of the total segregation sites (Table 4).

**Table 4:** Nucleotide variation within the *PtoSAHH *family in *Populus tomentosa *natural populations from three climatic regions

*Locus*	Climatic regions	*N*	*S*	*π*_tot_	*π*_sil_	*π*_s_	*π*_n_	*π*_n_/*π*_s_	Tajima's D*	Fu and Li's D*
*PtoSAHHA*	Northeastern region	14	82	0.01037	0.01721	0.02315	0.00236	0.10194	-0.05743	-0.97162
	Southern region	15	91	0.01073	0.01767	0.02229	0.00262	0.11754	-0.29863	-1.07951
	Northwestern region	14	83	0.00947	0.01583	0.02194	0.00202	0.09207	-0.43050	-1.03371
	Total	43	166	0.01058	0.01763	0.02419	0.00233	0.09632	-1.20660	-4.15376*

*PtoSAHHB*	Northeastern region	14	74	0.00961	0.01504	0.01580	0.00324	0.20506	0.01985	-0.66621
	Southern region	15	87	0.00959	0.01485	0.01400	0.00339	0.24214	-0.56229	-1.56176
	Northwestern region	14	68	0.00922	0.01429	0.01316	0.00325	0.24696	0.21416	-0.65261
	Total	43	160	0.00930	0.01444	0.01404	0.00326	0.23219	-1.42774	-3.95020*

N = Number of sequences sampled; S = number of segregating sites; π_tot _= average nucleotide diversity in the full-length gene; π_sil _= average nucleotide diversity in synonymous and noncoding sites, π_s _= average nucleotide diversity of synonymous mutations; π_n _= average nucleotide diversity of nonsynonymous mutations; *P <0.05.

**Figure 5:**
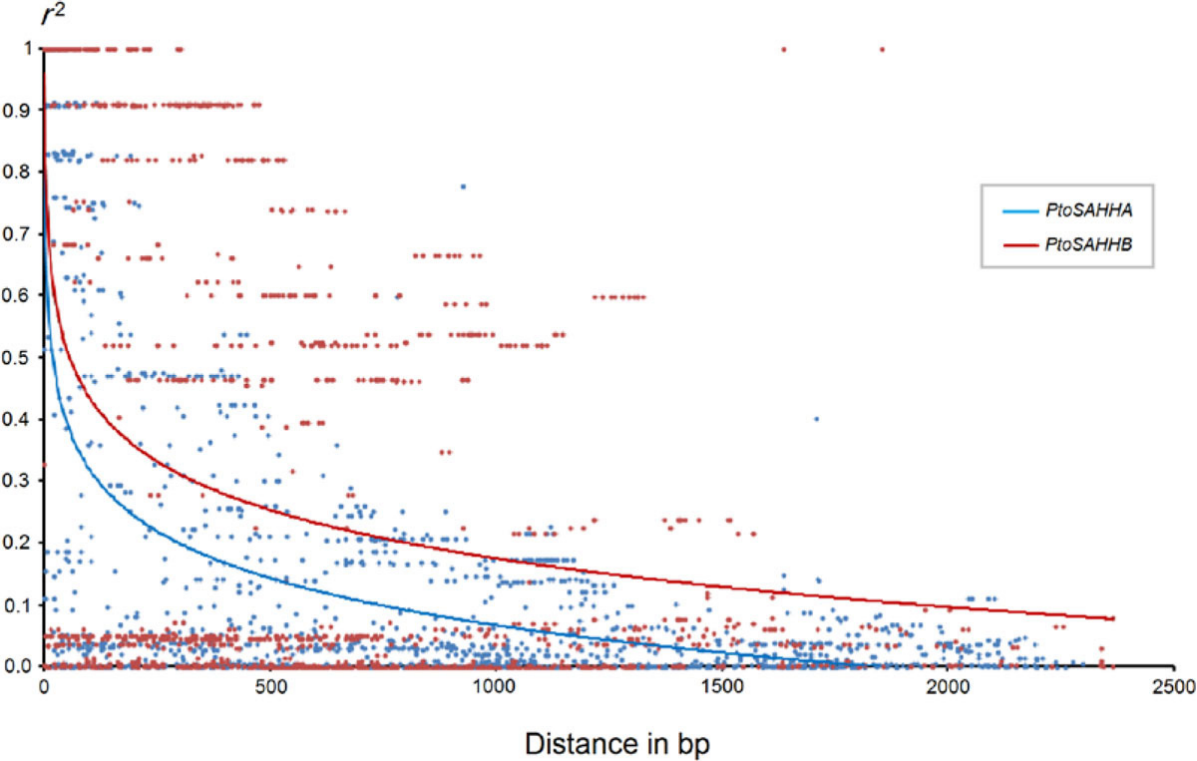
**Decay of linkage disequilibrium within *PtoSAHHA and PtoSAHHB *in the natural population**. Pairwise correlations between SNPs were plotted against the physical distance between SNPs (bp). Curves describe the nonlinear regression of *r*^2 ^(Er2) onto the physical distance (bp).

The nonlinear regression model for analyzing the decay of LD with distance showed that LD decayed quite rapidly with distance when total informative SNPs of *PtoSAHHA *and *PtoSAHHB *were used. However, LD decayed quickly within *PtoSAHHA*, with *r*^2 ^[26] dropping below 0.1 within ~800 bp (Figure 5), indicating that LD did not extend over the entire gene region. However, *PtoSAHHB *showed an extensive LD level over distance approaching the full length of the gene region (*r*^2 ^> 0.1, within 2,200 bp; Figure 5).

### Association analyses in *PtoSAHH *family members

In the association (discovery) population, 1,040 tests (104 SNPs × 10 traits) in *PtoSAHHA *and *PtoSAHHB *were conducted with 10^4 ^permutations using a mixed linear model (MLM). Results of single-marker associations for each of the 10 phenotypic traits are presented in Table S3 in Additional file 1. In total, 29 significant associations with 10 traits were identified at the threshold of *P *< 0.05 (Table S3 in Additional file 1). However, following correction for multiple testing with a significance level of *Q <*0.10, the total number of significant associations was reduced to eight (Table 5). These eight associations representing eight unique SNPs from the exon, intron, and 3'-UTR regions of *PtoSAHHA *and *PtoSAHHB*, were significantly associated with five wood traits, including α-cellulose, holocellulose, fiber length, tree height (H), and stem volume (V) (Table 5). The loci explained a small proportion of the phenotypic variance, ranging from 1.73% to 4.00% (Table 5). Of these markers, both *PtoSAHHB_1065 *from intron 1 and *PtoSAHHA_2203 *from the 3'-UTR showed significant association with α-cellulose content. Similarly, *PtoSAHHA_1196 *and *PtoSAHHA_1028 *from intron 1 were both significantly associated with holocellulose content, whereas *PtoSAHHB_618 *from exon 1 and *PtoSAHHA_1313 *from intron 1 showed significant association with H (Table 5). Among these eight SNPs in *PtoSAHHA *and *PtoSAHHB*, one represented synonymous substitution, two were nonsynonymous, and others were located in UTRs (Table 5). Silent SNPs were not considered as potential false positives a priori since they may affect transcript level and codon usage [27,28].

**Table 5:** Significant SNP associations identified in *PtoSAHHA *and *PtoSAHHB *using association-linkage analyses

Trait	Locus	Position	mutation	Association population (*N *= 460)			Linkage population (*N *= 1,200)			
					
				*P-*value	*Q-*value	*R*^2 ^(%)	*P-*value	*Q- *value	Alleles of parents ^1^	*R*^2 ^(%)
α-cellulose										
	*PtoSAHHB_1065*	Intron 1	[A : G]^nc^	0.0007	0.0299	4.00	0.0069	0.0893	[AA : AG]	2.82
	*PtoSAHHA_2203*	3'-UTR	[G : T]^nc^	0.0064	0.0675	1.76	0.0015	0.0490	[GT : GT]	3.60
Holocellulose										
	*PtoSAHHA_1196*	Intron 1	[C : T]^nc^	0.0056	0.0602	1.80	/	/	/	/
	*PtoSAHHA_1028*	Intron 1	[A : T]^nc^	0.0065	0.0675	1.73	0.0129	*Q >*0.10	[AT : TT]	1.55
Fiber length										
	*PtoSAHHB_410*	Exon 1	[G : A]^ns^	0.0001	0.0210	3.04	0.0013	0.0490	[GA : GA]	3.00
Tree height (H)										
	*PtoSAHHB_618*	Exon 1	[A : C]^s^	0.0004	0.0299	3.46	/	/	/	/
	*PtoSAHHA_1313*	Intron 1	[A : T]^nc^	0.0040	0.0521	1.94	0.0104	*Q >*0.10	[AT : AT]	0.98
Stem volume (V)										
	*PtoSAHHA_2021*	Exon 2	[A : T]^ns^	0.0018	0.0480	2.27	/	/	/	/

*R*^2 ^= percentage of phenotypic variance explained; *Q*-value = correction for multiple tests [FDR (*Q*) ≤ 0.10];nonsynonymous polymorphism (ns); synonymous polymorphism (s); noncoding polymorphism (nc); /, no data were identified in this study;

^1^Alleles of parents [female : male].

**Figure 6:**
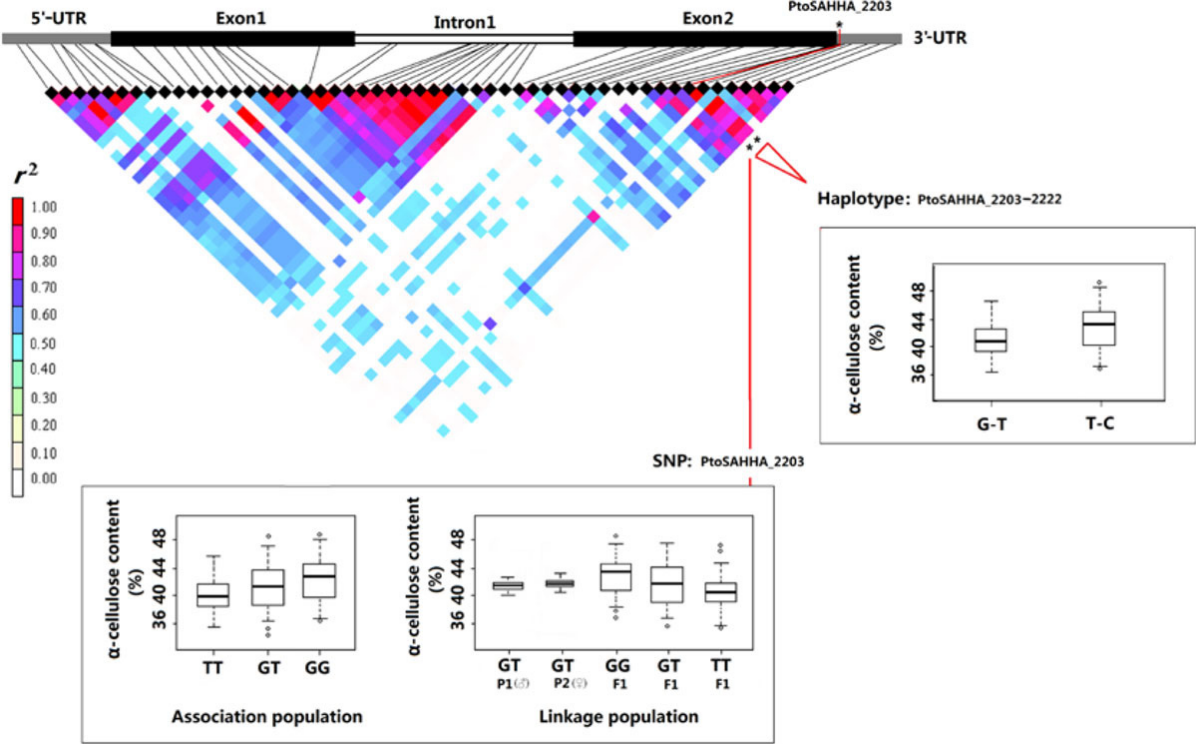
**Haplotype and single-marker associations with α-cellulose content for *PtoSAHHA***. Genotypic effect of the significant haplotype *PtoSAHHA_2203*-2222 (Q < 0.10) within *PtoSAHHA *is shown. The genotypic effect for single marker *PtoSAHHA_2203 *(Q < 0.10) is also revealed in both association and linkage populations.

**Figure 7:**
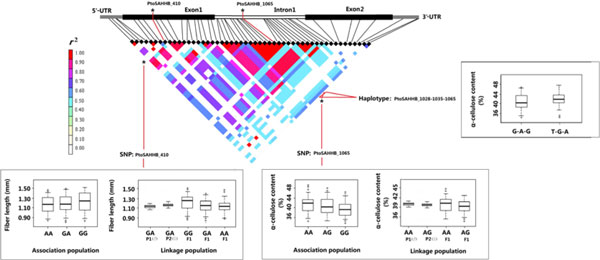
**Haplotype and single-marker associations with fiber length and α-cellulose content for *PtoSAHHB***. Effects of the significant haplotype (*Q *< 0.10) within *PtoSAHHB*. Marker effects of *PtoSAHHB_410 *and *PtoSAHHB_1065 *are also shown in both association and linkage populations. *PtoSAHHB_410 *is associated with fiber length, while *PtoSAHHB_1065 *is associated with α-cellulose content (*Q *< 0.1).

All eight significant SNPs identified in the discovery population were in accordance with Mendelian expectations (*P *≥ 0.01), and no novel allele was discovered in the linkage (validation) population. Consequently, 80 tests (8 SNPs × 10 traits) were conducted in the validation population, and five marker-trait associations were observed (*P *< 0.05; Table 5). After correcting for multiple testing (*Q <*0.10), only three significant markers were validated, including *PtoSAHHA_2203, PtoSAHHB_410*, and *PtoSAHHB_1065*, and the proportion of phenotypic variation was 3.60%, 3.00%, and 2.83%, respectively. Comparisons of genotypic effects for the same significant association examined in discovery and validation populations are shown in Figures 6 and Figure 7. As a result, the effects of different genotype classes in the noncoding markers *PtoSAHHB_1065 *(AA, AG) and *PtoSAHHA_2203 *(GG, GT, TT) were similar in both populations for α-cellulose content. The nonsynonymous marker *PtoSAHHB_410 *from exon 1 of *PtoSAHHB*, which results in an amino acid change from His to Arg, was significantly associated with fiber length. In addition, the effects of different genotype classes (GG, GA, AA) for fiber length were also similar in both populations (Figure 7). Moreover, *PtoSAHHB_410 *is located in a region of the SAHH protein that is predicted to be involved in an active functional domain.

To additionally dissect the allelic variations of the SNP identified in single-marker association analysis, we also tested the associations using a haplotype-based method in the discovery population. In total, 26 significant block sets (*r*^2 ^≥ 0.7, *P *< 0.0001) were analyzed with each of the 10 traits, and the number of common haplotypes (frequency ≥ 5%) per set varied from 2 to 6, with an average of 3.0. After multiple test corrections, eight significant blocks containing 14 significant haplotypes (*Q *< 0.10; Table S4 in Additional file 1) in *PtoSAHHA *and *PtoSAHHB *were associated with five traits, including α-cellulose content, holocellulose content, hemicellulose content, fiber width, diameter at breast height (DBH), and H, and many were strongly supported by single marker- association results (Tables 5 and S3). We also found that the haplotype block sizes for these significant SNPs were smaller in validation population than in the discovery population (Detail not shown).

## Discussion

### Characterization and function analysis of *SAHHs *in *Populus*

SAHH is a key enzyme in the maintenance of methylation potential in cells [12,29]. Inhibition of this enzyme causes increased accumulation of SAH, resulting in suppression of the methylation pathway via a feedback inhibition mechanism. In this study, two SAHHs encoded by *PtoSAHHA *and *PtoSAHHB *were determined to contain two active domains and a cofactor binding domain (NAD-binding domain; Figure 2), which is in accordance with the expected conserved features of SAHHs identified in other species. SAHHs belong to the larger family of NAD(P)H/NAD(P)^+^-binding proteins that share a Rossmann-fold, and the NAD(P)H/NAD(P)^+^-binding domain is found in numerous dehydrogenases as well as other redox enzymes, but is rather unusual for a hydrolase [30,31]. Therefore, the two functional domains (Figure 2) were predicted to catalyze the hydrolysis of SAH and thereby increase methylation efficiency [32].

In an early investigation, SAHH was found to be present in a cytokinin-binding protein complex isolated from tobacco leaves; therefore, the enzyme was proposed to be a cytokinin-binding protein [33]. Other studies demonstrated that downregulation of *SAHH *affected the expression of cytokinin pathway genes, and cytokinin positively regulated the transmethylation cycle and DNA methylation based on an analysis of a T-DNA mutant and transgenic RNAi plants [34]. Natural cytokinins are adenine derivatives that regulate numerous aspects of plant growth and development, stem growth and branching, leaf senescence, light signal transduction, and stress tolerance. Thus, *SAHH *appears to coexpress with cytokinin-related genes in plant growth and development. Xylogenesis is one of the most remarkable examples of irreversible plant cell differentiation. This process is controlled by a wide variety of factors both exogenous (photoperiod and temperature) and endogenous (phytohormones), and through an interaction between them [35,36]. The role of phytohormones in procambium initiation, cambial cell division, primary cell wall expansion, and secondary wall formation has been reviewed by Sundberg [37] and Mellerowicz [38]. Recent findings have demonstrated the existence of an auxin (indole-3-acetic acid, IAA) gradient across the developing vascular tissues of pine and poplar, and other hormones have been shown to be involved in xylogenesis by interacting with IAA in a synergetic (gibberellins, cytokinins, and ethylene) or inhibitory (abscisic acid) manner [39]. Consistently, *PtoSAHHs *from *P. tomentosa *may affect secondary cell wall formation by influencing the cytokinin content [33,40].

SAHH is one of the most highly conserved biosynthetic enzymes in the process of evolution [41], which is consistent with our finding that the two PtoSAHH proteins were in the same subgroup of the phylogenetic tree (Figure 3). This high level of sequence conservation is astonishing and highlights the important cellular function of the enzyme. Intracellular SAHH can regulate gene expression by affecting cytokinin content and DNA methylation status, thereby regulating plant growth and development [33,42]. In this study, *PtoSAHHA *and *PtoSAHHB *were originally isolated from a mature xylem cDNA library of *P. tomentosa*, and both were determined to share xylem-specific expression patterns (Figure 4), demonstrating that *PtoSAHHs *are likely associated with secondary cell wall development and may further participate in stem growth and wood formation.

### Dissecting allelic polymorphisms underlying growth and wood properties

Poplars are a model species for studies of angiosperm trees, provide data for comparison of a long-lived perennial to short-lived model plants (e.g., *Arabidopsis*, rice), but also offer new opportunities to explore the genetic basis of wood formation, perenniality, and dormancy [43,44]. Considering the important role of poplars, the identification of genes and allelic variants controlling growth and wood quality is important for forest tree breeding programs with a practical importance in production. Association mapping can detect functional allelic variation underlying quantitative traits, and these significant markers can be used for marker-assisted breeding. A set of candidate gene SNP associations was identified with chemical wood properties in related *Populus *species [45-47].

In this study, three single-marker associations and 14 haplotypes within *PtoSAHHs *were significantly associated with wood quality and growth traits (Tables 5 and S4), which demonstrate that *PtoSAHHs *may further participate in stem growth and wood formation. *PtoSAHHB_1065 *(located in intron 1 of *PtoSAHHB*) was significantly associated with α-cellulose content in both discovery and validation populations. Correspondingly, the significant haplotype-based associations (*PtoSAHHB_1028-1035-1065*) with α-cellulose in the discovery population suggest that this locus may be closely located to causative polymorphisms. This conjecture is supported by significant phenotypic differences in various genotype classes of *PtoSAHHB_1065 *in both populations (Figure 7). Consistently, *PtoSAHHA_2203 *(located in the 3'-UTR of *PtoSAHHA*), with two haplotype-based associations (*PtoSAHHA_2203-2222*), was also significantly associated with α-cellulose content in both populations. SNPs in noncoding regions (5′-UTR, 3'-UTR, and intron) could influence phenotypic traits because these regions play an important role in regulating gene expression. Specifically, SNPs in introns could affect phenotypic traits because those particular introns may play an important role in regulating gene expression and exon splicing; although mutation of the 3'-UTR did not result in an amino acid change, it may regulate expression of the gene; and SNPs in 5′-UTRs can affect mRNA stability, translational efficiency, or subcellular localization [48,49]. Previous studies have determined that SNP loci in noncoding regions are significantly associated with wood traits. For example, González-Martínez [19] detected a strong association between SNP M10, located in intron 1, and earlywood microfibril angle in *Pinus taeda*. Fang [50] detected a novel SNP in the 3' flanking region of the goat *BMP-2 *gene, which is associated with growth traits. Similarly, an SNP in the 5'-UTR of *Eni-HB1 *associated with microfibril angle was identified in *Eucalyptus nitens *[51]. In addition, two SNPs located in the 5'-UTR of *TUB15 *were associated with lignin content in *Populus nigra *[52].

A nonsynonymous substitution in exon 1 of *PtoSAHHB *(*PtoSAHHB_410*) was strongly associated with fiber length using single-marker association. No haplotype was found there, demonstrating that *PtoSAHHB_410 *is a unique functional locus. The G allele is the minor allele of this nonsynonymous marker, which represents a missense mutation causing a His→Arg substitution. Fibers, the most abundant secondary wall-containing cells in woody species, are mainly controlled by the endogenous regulation of cell elongation and expansion [53-55]. During secondary wall formation, highly coordinated expression of multiple genes controls cell elongation and secondary wall thickening of fibers [56-58]. For example, a mutant allele of *AtCesA7 *in fragile fiber 5 (fra5) causes a severe decrease in cellulose content and fiber thickness [58]. *AtCesA7*/*IRX3 *and *AtCOBL4*/*IRX6 *are coexpressed in tissues during secondary cell wall development, and loss-of-function mutants of either of these genes show diminished cellulose content and loss of mechanical strength of the plant body [58]. From the results described above, we inferred that *PtoSAHHB_410 *may be a functional mutation that is in or near a causative locus involved in fiber morphology. Further analysis of the protein structure encoded by *PtoSAHHB *revealed that the nonsynonymous mutation of amino acid 94 (His→Arg) is within the SAHH signature motifs (at residues 85-99) and close to the putative transmembrane domains (TMDs; at residues 63-86; Figure 2), suggesting that this nonsynonymous locus may affect the enzymatic activity of SAHH signature motifs and also influence gene expression related to fiber length. Therefore, expanding our understanding of the action of *PtoSAHHB *is essential.

Wood formation mainly includes deposition of strong secondary cell walls that contain cellulose microfibrils, lignin, and other components. Many studies have examined the molecular biology of secondary cell wall biosynthesis and have shown that the complex, dynamic process of secondary wall formation requires the coordinate regulation of diverse metabolic pathways involving polysaccharides and lignin. Furthermore, the incorporation of association studies by using more genes in shared biosynthetic pathways or the whole genome-wide level would provide a more complete dissection of genetic variance for the growth and lignocellulosic traits. The finding can be applied to marker-assisted breeding.

## Conclusions

SAHH is a key enzyme in the maintenance of methylation potential in cells, and can further affect plant growth and development. This study first identified *SAHH *family (*PtoSAHHA *and *PtoSAHHB*) from *P. tomentosa*, and the high level of sequence conservation of encoded proteins indicated the crucial function of the SAHH family. Phylogenetic analyses demonstrated that all plant SAHHs were split off before the divergence of monocots and dicots ~200 million years ago, and the *PtoSAHH *members were split off prior to the divergence of interspecies in *Populus*. Tissue-specific expression profiles of the *PtoSAHH *family revealed similar expression patterns, with high expression in the xylem, indicating putative functional roles in wood formation. Subsequently, single-marker and haplotype-based association tests (using a discovery population), as well as linkage analyses for validation, demonstrated two noncoding SNPs and corresponding haplotypes that were remarkably associated with the α-cellulose content; one nonsynonymous SNP showed significant association with fiber length. We inferred that the nonsynonymous SNP (PtoSAHHB_410) may be a functional mutation that is in or near a causative locus involved in fiber morphology. In conclusion, the present study offers a theoretical basis for better understanding the regulatory mechanism of the *PtoSAHH *family in secondary cell wall formation.

## Methods

### Plant materials and phenotypic data

*Discovery population*: In 1982, a clonal arboretum of *P. tomentosa *was established in Guan Xian County, Shandong Province, China (36°23′N, 115°47′E), which contained 1,047 unrelated individuals from the entire nature distribution region (~1 million km^2^) of *P. tomentosa*. The distribution zone can be divided into three climatic regions: Southern (S), Northwestern (NW), and Northeastern (NE), by the methods of principal components analysis and isodata fuzzy cluster of 16 meteorological factors [59]. Unrelated *P. tomentosa *individuals were randomly selected from the clonal arboretum for identifying SNPs and association studies (43 and 460, respectively).

*Validation population*: In 2008, 5,000 F_1 _hybrid progeny established by controlled crossing between two elite poplar parents, clone "YX01" (*P. alba *× *P. glandulosa*; female) and clone "LM 50" (*P. tomentosa*; male), were grown in the Xiao Tangshan horticultural fields of Beijing Forestry University, Beijing, China (40°2′N, 115°50′E). For future validation of significant associations identified in a discovery population, 1,200 individuals were randomly selected from 5,000 F_1 _progeny, which composed the validation population.

*Phenotypic data*: In discovery and validation populations, 10 quantitative phenotypic traits were scored with at least three ramets per genotype. These 10 traits included growth characteristics (H, DBH, and V) and wood properties (fiber length, fiber width, microfiber angle, holocellulose, hemicelluloses, α-cellulose, and lignin contents), and the distributional values of each trait were approximately consistent with a normal distribution. Details of the sampling and measurement methods, phenotypic variance, and Pearson's correlations for these 10 traits have been reported previously [47,60].

### Isolation of *PtoSAHHA *and *PtoSAHHB *cDNAs

Using the Plant Qiagen RNeasy kit, RNA from the mature xylem stem tissue of a *P. tomentosa *(clone "*LM50*"; 1-year-old) was extracted and then reverse transcribed into cDNA with the SuperScript First-Strand Synthesis system (Life Technologies, Carlsbad, CA, USA). The *P. tomentosa *stem mature xylem cDNA library was constructed, which was generated as a part of our large-scale effort to identify genes expressed predominantly in the mature xylem of *P. tomentosa *stems. The cDNA library was composed of 5.0 × 10^6 ^pfu with an insert size of 1.0-4.0 kb. Subsequently, random end-sequencing of 5,000 cDNA clones and comparison with all available *Arabidopsis SAHH *sequences revealed that 10 clones were highly similar to *AtSAHH*. Finally, with these expressed sequence tag (EST) sequences, one contig was assembled representing a full-length cDNA. Next, the BLAST program (JGI database) was used to analyze the ESTs. Two full-length cDNAs of *SAHH *were detected from *P. trichocarpa*. Based on these two cDNAs, gene-specific primers were designed and two full-length cDNAs of *SAHH *from *P. tomentosa *were isolated (*PtoSAHHA *and *PtoSAHHB*).

### DNA extraction and *SAHH *genomic DNA identification

Using the Plant DNeasy kit, total genomic DNA was extracted from fresh young leaves of each individual *P. tomentosa *in accordance with the manufacturer's protocol (Life Technologies). For sequencing the genomic DNA of *PtoSAHH*, specific primers were designed based on the two cDNA sequences. PCR amplification was performed according to the procedure described by Du [61]. Next, PCR products were resolved by agarose gel electrophoresis, excised, and purified using Ultrafree^®^-DA (Millipore, Billerica, MA, USA) centrifugal filter units. Purified DNA was then ligated into the pGEM^®^-T Easy Vector and transformed into JM109 competent cells (Promega, Madison, WI, USA). Plasmid DNA was isolated from overnight cultures using the QIAprep Spin Miniprep protocol (Qiagen, Valencia, CA, USA) and sequenced on both strands with conserved T7 and SP6 primers using the BigDye™ Terminator Cycle Sequencing Kit (version 3.1; Applied Biosystems, Foster City, CA, USA) and a 4300 DNA Analyzer (Li-Cor Biosciences, Lincoln, NE, USA).

## Gene structure and phylogenetic analysis

The Gene Structure Display Server (GSDS) program (http://gsds.cbi.pku.edu.cn/) was used to represent the gene structure schematic diagrams of *PtoSAHHA *and *PtoSAHHB *after submitting coding and genomic sequences.

Multiple sequence alignments and an unrooted phylogenetic tree of the amino acid sequences of *SAHH *in monocotyledons, dicotyledons, and algae were generated using the NJ method of MEGA version 5.05, and statistical confidence of the tree nodes was based on 1,000 bootstrap replicates. *SAHH *gene sequences in *Arabidopsis, P. trichocarpa*, rice, maize, and cotton were identified by searching public databases available at NCBI (http://www.ncbi.nlm.nih.gov) [62].

### Tissue-specific expression analysis

Total RNA was extracted from at least three individual samples of all fresh tissues (root, stem phloem, stem cambium, stem immature xylem, stem mature xylem, young leaf, mature leaf, and apical shoot meristem) collected from a 1-year-old *P. tomentosa *clone, "LM50." Additionally, RNA was extracted using the Plant Qiagen RNAeasy Kit according to the manufacturer's instructions (Qiagen). Purified RNA was treated with DNaseI using the RNase-Free DNase set (Qiagen). Finally, RNA integrity was confirmed on an agarose gel. RNA was then reverse transcribed into cDNA using the SuperScript First-Strand synthesis system and the supplied polythymine primers (Invitrogen, Carlsbad, CA, USA) [63]. All cDNA samples were used for testing tissue-specific expression of *PtoSAHHA *and *PtoSAHHB*.

Using the *PtoSAHH*-specific and internal control (*Actin*) primer pairs designed by Primer Express 3.0 software (Applied Biosystems), the cDNA (2 μL) of all fresh tissues was amplified in a reaction containing 12.5 μL of QuantiTect SYBR Green PCR reagent (Qiagen), 0.5 μL each of 10 nM forward and reverse primers, and 9.5 μL of water. Amplification was performed on a 7500 Fast Real-Time PCR System (Applied Biosystems). Real-time quantitative PCR and the generated real-time data were performed according to the procedure described by Zhang [63]. All reactions were performed in triplicate for technical and triplicate biological repetitions of three plants, respectively, and the results were standardized to *actin*.

### Nucleotide diversity and linkage disequilibrium

To identify SNPs within *PtoSAHHA *and *PtoSAHHB*, the two full-length genes were sequenced and analyzed in 43 unrelated individuals from the discovery population. Multiple sequence alignment was analyzed using DNA sequence polymorphism (DNASP) software version 5.10 [64]. Insertions and deletions (indels) were excluded from all estimates. Next, 78 common SNPs (minor allele frequencies ≥ 0.05, 42 SNPs from *PtoSAHHA *and 36 from *PtoSAHHB*) were genotyped by the single-nucleotide primer extension method with a Beckman Coulter (Franklin Lakes, NJ, USA) sequencing system across all DNA samples.

Additionally, DNASP software version 5.10 was used to calculate summary statistics for nucleotide diversity and divergence. Nucleotide diversity was estimated by θw from the number of polymorphic segregating sites [65,66], and by π from the number of pairwise differences per site between sequences [66]. In addition, the diversity statistics of noncoding, synonymous, and nonsynonymous sites, and neutrality test statistics, Tajima's D* [24], and Fu and Li's D* [25] of three climatic regions were also calculated. To estimate if natural selection (purifying selection or positive selection) is involved in evolving this enzyme during species speciation, we do dN/dS analysis (between species) with all homologous DNA sequences data from different species (Table S1 in Additional file 1).

LD descriptive statistics (*r*^2^) are affected by both recombination and differences in allele frequencies between sites [26]. To assess the extent of LD within the sequenced *PtoSAHHA *and *PtoSAHHB *regions, the decay of LD with physical distance (base pairs) between informative SNPs within genes was estimated by nonlinear regression analysis [67]. Singletons were excluded in LD analyses, and the significance level for LD was determined through 10,000 permutations.

### Association tests

*SNP association models*: Associations between 10 traits and 78 common SNP markers of *PtoSAHH *(42 from *PtoSAHHA *and 36 from *PtoSAHHB*) in the discovery population (460 individuals) were tested via the MLM implemented in TASSEL ver. 2.0.1. The MLM can be described as follows: *y *= *µ *+*Qv+ Zu *+ *e*, where *y *is a vector of phenotype observation, *µ *is a vector of intercepts; *v *is a vector of population effects; *u *is a vector of random polygene background effects; *e *is a vector of random experimental errors; *Q *is a matrix defining the population structure, and *Z *is a matrix relating *y *to *u*. For Var (*u*) = G =*σ^2^_a_K *with *σ^2^_a _*as the unknown additive genetic variance and *K *as the kinship matrix [68]. In the MLM model, the kinship matrix was built using the SPAGeDi version 1.2 software [69], and the population structure matrix was identified based on significant subpopulations [70]. Failure to appropriately adjust for multiple testing may produce excessive false positives or overlook true positive signals in association studies when using large numbers of SNPs. To correct for multiple tests, the positive false discovery rate (FDR) method was used to identify significant SNPs after correction using QVALUE software, version 1.0 [71].

Subsequently, all eight significant SNPs (*Q *< 0.10) identified in the discovery population were genotyped in the validation population for confirmation. Inheritance tests of all SNPs were first examined in the validation population with 1,200 individuals by performing a chi-square (*χ*^2^) test (0.01 probability), and SNPs following Mendelian expectations (*P *≥ 0.01) were then used in the single-marker analysis in validation population (excluding the genotype data involving null alleles at each locus). Significant SNPs were calculated by PLINK version 1.07 [72], and the FDR method was used to perform a correction for multiple testing

*Haplotype-based association analysis*: Haplotypes were inferred and haplotype-based association tests with growth and wood quality were performed using haplotype trend regression software [73]. Haplotype association significance was based on 1,000 permutation tests. Singleton alleles and haplotypes with a frequency <5% were ignored when constructing the haplotypes. A correction for multiple tests was performed using the positive FDR method.

## Competing interests

The authors declared that they have no competing interests.

## Authors' contributions

Conceived and designed the experiments: DZ. Performed the experiments: LW DZ QD WP HY CG. Analyzed the data: QD DZ WP CG LW. Contributed reagents/materials/analysis tools: QD DZ LW DZ. Wrote the paper: QD WP DZ.

## Funding

Publication of this work was supported by grants from the State Key Basic Research Program of China (No. 2012CB114506), and Program for Changjiang Scholars and Innovative Research Team in University (No. IRT13047), and the Projects of the National Natural Science Foundation of China (No. 31170622, 30872042).

## Supplementary Material

Additional file 1 Table S1 SAHH protein sequences from species used in this study. Table S2 Primers used for real-time PCR analysis. Table S3 Significant SNP associations (*P *≤ 0.05) identified in *PtoSAHHA *and *PtoSAHHB*. Table S4 List of significant haplotype-based associations with wood quality and growth traits in the *Populus tomentosa *association population (n = 460). Click here for file

## Notes

This article has been published as part of *BMC Genetics *Volume 15 Supplement 1, 2014: Selected articles from the International Symposium on Quantitative Genetics and Genomics of Woody Plants. The full contents of the supplement are available online at http://www.biomedcentral.com/bmcgenet/supplements/15/S1.

## References

[B1] Luka Z Mudd SH Wagner C Glycine N-methyltransferase and regulation of S-adenosylmethionine levels J Biol Chem 2009 284 22507 22511 10.1074/jbc.R109.019273 19483083 PMC2755656

[B2] Moffat BA Weretilnyk EA Sustaining S-adenosyl-L-methionine-dependent methyltransferase activity in plant cells Physiol Plant 2001 113 435 442 10.1034/j.1399-3054.2001.1130401.x

[B3] de la Haba Cantoni G The enzymatic synthesis of S-adenosyl-L-homocysteine from adenosine and homocysteine J Biol Chem 1959 234 603 608 13641268

[B4] Ericson LE Williams JN Elvehjem C Enzymatic cleavage of S-adenosylhomocysteine and the transfer of labile methyl groups Acta Chem. Scand 1955 9 859 860

[B5] Baddiley J Jamieson G A G A Synthesis of S-(5′-deoxyadenosine-5′)-homocysteine, a product from enzymic methylations involving "active methionine" J Chem Soc 1955 1085 1089

[B6] Cantoni GL Scarano E The formation of S-adenosylhomocysteine in enzymatic transmethylation reaction J Am Chem Soc 1954 76 4744

[B7] Porcelli M Fusco S Inizio T Zappia V Cacciapuoti G Expression, purification, and characterization of recombinant S-Adenosylhomocysteine Hydrolase from the thermophilic archaeon *Sulfolobus solfataricus* Protein Expres Purif 2000 18 27 35 10.1006/prep.1999.1161 10648166

[B8] Parker NB Yang X Hanke J Mason KA Schowen RL Trypanosoma cruzi: molecular cloning and characterization of the S-adenosylhomocysteine hydrolase Exp Parasitol 2003 105 2 149 158 10.1016/j.exppara.2003.10.001 14969692

[B9] Tehlivets O Hasslacher M Kohlwein S S-adenosyl-L-homocysteine hydrolase in yeast: key enzyme of methylation metabolism and coordinated regulation with phospholipid synthesis FEBS Lett 2004 577 3 501 506 10.1016/j.febslet.2004.10.057 15556636

[B10] She YB Zhu YC Zhang TZ Guo WZ Cloning, expression, and mapping of S-adenosyl-L-homocysteine hydrolase (*GhSAHH*) cDNA in cotton Acta Agron Sin 2008 34 6 958 964 10.3724/SP.J.1006.2008.00958

[B11] Jin XX Qin ZW ZHou XY Wu T Cloning and Expression Analysis of S-Adenosyl-L-Homocysteine Hydrolase in Cucumber (*Cucumis stavius *L.) Acta Agron Sin 2012 45 7 1338 1346

[B12] Tanaka H Masuta C Uehara K Kataoka J Koiwai A Noma M Morphological changes and hypomethylation of DNA in transgenic tobacco expressing antisense RNA of the Sadenosyl-L-homocysteine hydrolase gene Plant Mol Biol 1997 35 981 986 10.1023/A:1005896711321 9426618

[B13] Rocha PS Sheikh M Melchiorre R Fagard M Boutet S Loach R Moffatt B Wagner C Vaucheret H Furner I The Arabidopsis HOMOLOGY-DEPENDENT GENE SILENCING1 gene codes for an S-adenosyl-L-homocysteine hydrolase required for DNA methylation-dependent gene silencing Plant Cell 2005 17 404 417 10.1105/tpc.104.028332 15659630 PMC548815

[B14] Godge MR Kumar D Kumar PP Arabidopsis HOG1 gene and its petunia homolog PETCBP act as key regulators of yield parameters Plant Cell Rep 2008 27 1497 1507 10.1007/s00299-008-0576-z 18592247

[B15] Collard B Mackill D Marker-assisted selection: an approach for precision plant breeding in the twenty-first century Phil Trans R Soc B 2008 363 557 572 10.1098/rstb.2007.2170 17715053 PMC2610170

[B16] Neale DB Kremer A Forest tree genomics: growing resources and applications Nat Rev Genet 2011 12 111 122 10.1038/nrg2931 21245829

[B17] Zhang DQ Zhang ZY Single nucleotide polymorphisms discovery and linkage disequilibrium For Studies China 2005 7 1 14

[B18] Eckert AJ Bower AD Wegrzyn JL Pande B Jermstad KD Krutovsky KV Clair JBS Neale DB Association genetics of coastal douglas fir (*Pseudotsuga menziesu *var. menziesii, Pinaceae) Genetics 2009 182 1289 1302 I. Cold-hardiness related traits. 10.1534/genetics.109.102350 19487566 PMC2728866

[B19] González-Martínez SC Wheeler NC Ersoz E Nelson CD Neale DB Association genetics in *Pinus taeda *L. I. Wood property traits Genetics 2007 175 399 409 17110498 10.1534/genetics.106.061127 PMC1775017

[B20] González-Martínez SC Huber D Ersoz E Davis JM Neale DB Association genetics in *Pinus taeda *L. II. Carbon isotope discrimination Heredity 2008 101 19 26 18478029 10.1038/hdy.2008.21

[B21] Wegrzyn JL Eckert AJ Choi M Lee JM Stanton BJ Sykes R Davis MF Tsai CJ Neale DB Association genetics of traits controlling lignin and cellulose biosynthesis in black cottonwood (*Populus trichocarpa*, Salicaceae) secondary xylem New Phytol 2010 188 515 532 10.1111/j.1469-8137.2010.03415.x 20831625

[B22] Dillon SK Brawner JT Meder R Lee DJ Southerton SG Association genetics in *Corymbia citriodora *subsp. Variegate identifies single nucleotide polymorphisms affecting wood growth and cellulosic pulp yield New Phytol 2012 195 596 608 10.1111/j.1469-8137.2012.04200.x 22680066

[B23] Mitchell-Olds T Clauss MJ Plant evolutionary genomics Curr Opin Plant Biol 2002 5 74 79 10.1016/S1369-5266(01)00231-X 11788312

[B24] Tajima F Statistical method for testing the neutral mutation hypothesis by DNA polymorphism Genetics 1989 123 3 585 595 2513255 10.1093/genetics/123.3.585 PMC1203831

[B25] Fu Y Li W Statistical tests of neutrality of mutatons Genetics 1993 133 3 693 709 8454210 10.1093/genetics/133.3.693 PMC1205353

[B26] Hill WG Robertson A Linkage disequilibrium in finite populations Theor Appl Genet 1968 38 226 231 10.1007/BF01245622 24442307

[B27] Kimchi-Sarfaty C Oh JM Kim IW Sauna ZE Calcagno AM Ambudkar SV Gottesman MM A "silent" polymorphism in the *MDR1 *gene changes substrate specificity Science 2007 315 525 528 10.1126/science.1135308 17185560

[B28] Chamary HV Hurst LD The price of silent mutations Sci Am 2009 300 6 46 53 10.1038/scientificamerican0609-46 19485088

[B29] Miller MW Duhl DM Winkes BM Arredondo-Vega F Saxon PJ Wolff GL Epstein CJ Hershfield MS Barsh GS The mouse lethal nonagouti (a(x)) mutation deletes the S-adenosylhomocysteine hydrolase (Ahcy) gene EMBO J 1994 13 1806 1816 8168479 10.1002/j.1460-2075.1994.tb06449.x PMC395020

[B30] Rao ST Rossmann MG Comparison of super-secondary structures in proteins J Mol Biol 1973 76 241 256 10.1016/0022-2836(73)90388-4 4737475

[B31] Hoffman DR Marion DW Cornatzer WE Duerre JA S-Adenosylmethionine andS-adenosylhomocysteine metabolism in isolated liver J Biol Chem 1980 22 10822 10827 7430157

[B32] Hermes M Osswald H Mattar J Kloor D Influence of an altered methylation potential on mRNA methylation and gene expression Exp Cell Res 2004 294 325 334 10.1016/j.yexcr.2003.12.001 15023523

[B33] Masuta C Tanaka H Uehara K Kuwata S Koiwai A Noma M Broad resistance to plant viruses in transgenic plants conferred by antisense inhibition of a host gene essential in *S*-adenosylmethionine-dependent transmethylation reactions Proc Natl Acad Sci USA 1995 92 6117 6121 10.1073/pnas.92.13.6117 11607550 PMC41653

[B34] Li CH Yu N Jiang SM Shangguan XX Wang LJ Chen XY Down-regulation of *S*-adenosyl-L-homocysteine hydrolase reveals a role of cytokinin in promoting transmethylation reactions Planta 2008 228 125 136 10.1007/s00425-008-0724-2 18350315

[B35] Mitsui S Wakasugi T Sugiura M A cDNA encoding the 57 kDa subunit of a cytokinin-binding protein complex from tobacco: the subunit has high homology to *S*-adenosyl-L-homocysteine hydrolase Plant Cell Physiol 1993 34 1089 1096

[B36] Persson S Wei H Milne J Page GP Somerville CR Identification of genes required for cellulose synthesis by regression analysis of public microarray data sets Proc Natl Acad Sci USA 2005 102 8633 8638 10.1073/pnas.0503392102 15932943 PMC1142401

[B37] Somerville C Cellulose synthesis in higher plants Annu Rev Cell Dev Biol 2006 22 53 78 10.1146/annurev.cellbio.22.022206.160206 16824006

[B38] Sundberg B Uggla C Tuominen H Savidge, J Barnett, R Napier, eds. Cell and Molecular Biology of Wood Formation. "Cambial growth and auxin gradient." 2000 Oxford, BIOS Scientific Publishers Ltd, Oxford 169 188

[B39] Mellerowicz EJ Baucher M Sundberg B Boerjan W Unravelling cell wall formation in the woody dicot stem Plant Mol Biol 2001 47 239 274 10.1023/A:1010699919325 11554475

[B40] Plomion C Leprovost G Stokes A Wood formation in trees Plant physiol 2001 127 4 1513 1523 10.1104/pp.010816 11743096 PMC1540185

[B41] Mushegian AR Garey JR Martin J Liu LX Large-scale taxonomic profiling of eukaryotic model organisms: a comparison of orthologous proteins encoded by the human, fly, nematode, and yeast genomes Genome Res 1998 8 6 590 598 9647634 10.1101/gr.8.6.590

[B42] Li CH. Study on separation and cloning of cotton fiber cell elongation-related genes and their expression characteristics by cDNA-microarray MS Dissertation of Nanjing Agri Univ 2002

[B43] Brunner AM Busov VB Strauss SH Poplar genome sequence: functional genomics in an ecologically dominant plant species Trends Plant Sci 2004 9 49 56 14729219 10.1016/j.tplants.2003.11.006

[B44] Li L Lu S Chiang VL A genomic and molecular view of wood formation Crit Rev Plant Sci 2006 25 213 233

[B45] Du Q Pan W Tian J Li B Zhang D The UDP-Glucuronate Decarboxylase Gene Family in *Populus*: Structure, Expression, and Association Genetics PLoS ONE 2013 8 4 e60880. doi:10.1371/journal.pone.0060880 10.1371/journal.pone.0060880 23613749 PMC3629030

[B46] Guerra FP Wegrzyn JL Sykes R Davis MF Stanton BJ Neale DB. Association genetics of chemical wood properties in black poplar (*Populus nigra*) New Phytol 2013 197 162 176 10.1111/nph.12003 23157484

[B47] Tian J Du Q Chang M Zhang D Allelic Variation in PtGA20Ox Associates with Growth and Wood Properties in *Populus *spp PLoS ONE 2012 7 12 e53116 doi:10.1371/journal.pone.0053116 10.1371/journal.pone.0053116 23300875 PMC3534044

[B48] Vilela C McCarthy JEG Regulation of fungal gene expression via short open reading frames in the mRNA 5′untranslated region Mol microbiol 2003 49 859 867 10.1046/j.1365-2958.2003.03622.x 12890013

[B49] Lin Z Li WH Evolution of 5' untranslated region length and gene expression reprogramming in yeasts Mol Biol Evol 2012 29 1 81 89 10.1093/molbev/msr143 21965341 PMC3245540

[B50] Fang X Xu H Zhang C Zhang J Lan X Gu C Polymorphisms in BMP-2 gene and their associations with growth traits in goats Genes Genom 2010 32 29 35 10.1007/s13258-010-0762-6

[B51] Southerton SG MacMillan CP Bell JC Bhuiyan N Dowries G Ravenwood IC Joyce KR Williams D Thumma BR Association of allelic variation in xylem genes with wood properties in *Eucalyptus nitens* Austral For 2010 73 4 259 264 10.1080/00049158.2010.10676337

[B52] Guerra F Wegrzyn J Sykes R Davis M Stanton B Neale D Association genetics of chemical wood properties in black poplar (*Populus nigra*) New Phytol 2012 197 162 176 23157484 10.1111/nph.12003

[B53] Horn R Morphology of pulp fiber from hardwoods and influence on paper strength 1978 USDA For Serv Res Pap FPL 312, For Prod Lab, Madison, WI, USA,

[B54] Amidon TE Effect of the wood properties of hardwoods on kraft paper properties Tappi J 1981 64 123 126

[B55] Migneault S Koubaa A Erchiqui F Chaala A Englund K Krause C Wolcott M Effect of fiber length on processing and properties of extruded wood-fiber/HDPE composites J Appl Polym Sci 2008 110 1085 1092

[B56] Tanaka K Murata K Yamazaki M Onosato K Miyao A Hirochika H Three distinct rice cellulose synthase catalytic subunit genes required for cellulose synthesis in the secondary wall Plant Physiol 2003 133 73 83 10.1104/pp.103.022442 12970476 PMC196581

[B57] Burton RA Shirley NJ King BJ Harvey AJ Fincher GB The *CesA *gene family of barley (*Hordeum vulgare*): quantitative analysis of transcripts reveals two groups of co-expressed genes Plant Physiol 2004 134 224 236 10.1104/pp.103.032904 14701917 PMC316302

[B58] Zhong R Demura T Ye ZH SND1, a NAC domain transcription factor, is a key regulator of secondary wall synthesis in fibers of *Arabidopsis* Plant Cell 2006 18 3158 3170 10.1105/tpc.106.047399 17114348 PMC1693950

[B59] Huang ZH The study on the climatic regionalization of the distributional region of *Populus tomentosa* J Beijing For Univ 1992 14 26 32

[B60] Du Q Pan W Xu B Li B Zhang D Polymorphic simple sequence repeat (SSR) loci within cellulose synthase (*PtoCesA*) genes are associated with growth and wood properties in *Populus tomentosa* New Phytol 2013 197 763 776 10.1111/nph.12072 23278184

[B61] Du Q Zhang D Li B Development of 15 novel microsatellite markers from cellulose synthase genes in *Populus tomentosa *(Salicaceae) Am J Bot 2012 99 e46 e48 10.3732/ajb.1100308 22268219

[B62] Altschul SF Madden TL Schäffer AA Zhang J Zhang Z Miller W Lipman DJ Gapped BLAST and PSI-BLAST: a new generation of protein database search programs Nucleic Acids Res 1997 25 17 3389 3402 10.1093/nar/25.17.3389 9254694 PMC146917

[B63] Zhang D Du Q Xu B Zhang Z Li B The actin multigene family in *Populus*: organization, expression and phylogenetic analysis Mol Genet Genomics 2010 284 105 119 10.1007/s00438-010-0552-5 20577761

[B64] Rozas J Sa´nchez-Delbarrio JC Messeguer X Rozas R DnaSP, DNA polymorphism analyses by the coalescent and other methods Bioinformatics 2003 19 2496 2497 10.1093/bioinformatics/btg359 14668244

[B65] Watterson G On the number of segregating sites in genetical models without recombination Theor Popul Biol 1975 7 188 193 10.1016/0040-5809(75)90020-9 1145509

[B66] Nei M Molecular evolutionary genetics 1987 Columbia University Press, New York

[B67] Remington DL Thornsberry JM Matsuoka Y Wilson LM Whitt SR Doebley J Kresovich S Goodman MM Buckler ES Structure of linkage disequilibrium and phenotypic associations in the maize genome Proc Natl Acad Sci USA 2001 98 11479 11484 10.1073/pnas.201394398 11562485 PMC58755

[B68] Bradbury PJ Zhang Z Kroon DE Casstevens TM Ramdoss Y Buckler ES TASSEL: software for association mapping of complex traits in diverse samples Bioinformatics 2007 23 2633 2635 10.1093/bioinformatics/btm308 17586829

[B69] Hardy OJ Vekemans X SPAGEDi: a versatile computer program to analyze spatial genetic structure at the individual or population levels Mol Ecol Notes 2002 2 618 620 10.1046/j.1471-8286.2002.00305.x

[B70] Du Q Wang B Wei Z Zhang D Li B Genetic diversity andpopulation structure of Chinese white poplar (*Populus tomentosa*) revealed by SSR markers J Hered 2012 103 853 862 10.1093/jhered/ess061 23008443

[B71] Storey J Tibshirani R Statistical significance for genome wide studies Proc Natl Acad Sci USA 2003 100 9440 9445 12883005 10.1073/pnas.1530509100 PMC170937

[B72] Purcell S Neale B Todd-Brown K Thomas L Ferreira M Bender D Maller J Sklar P de Bakker P Daly M Sham P PLINK:a tool set for whole-genome association and population-based linkage analyses Am J Hum Genet 2007 81 3 559 575 10.1086/519795 17701901 PMC1950838

[B73] Higuchi T Biochemistry and molecular biology of wood 1997 Springer Verlag, London

