# Supplementary material for: Allelic variation within the S-adenosyl-L-homocysteine hydrolase gene family is associated with wood properties in Chinese white poplar (Populus tomentosa)
Source: BMC Genet. 2014 Jun 20;15(Suppl 1):S4. doi: 10.1186/1471-2156-15-S1-S4 (PMC4118623; doi:10.1186/1471-2156-15-S1-S4)
Supplement: Additional file 1 — Table S1 SAHH protein sequences from species used in this study. Table S2 Primers used for real-time PCR analysis. Table S3 Significant SNP associations (P ≤ 0.05) identified in PtoSAHHA and PtoSAHHB. Table S4 List of significant haplotype-based associations with wood quality and growth traits in the Populus tomentosa association population (n = 460). [file 1471-2156-15-S1-S4-S1.zip › Additional file 1/Table S1.doc]

Table S1 SAHH protein sequences from species used in this study

|  | Origin | Function | GenBank Accession No. |
| --- | --- | --- | --- |
| Dicotyledons |  |  |  |
|  | *Arabidopsis thaliana* (At) | *S*-adenosyl-l-homocysteine hydrolase | AAP92453.1 |
|  | *Brassica oleracea* (Bo) | *S*-adenosyl-l-homocysteine hydrolase | AAP92454.1 |
|  | *Capsicum annuum* (Ca) | *S*-adenosyl-l-homocysteine hydrolase | AFU51537.1 |
|  | *Chrysanthemum* × *morifolium* (Cm) | *S*-adenosyl-l-homocysteine hydrolase | ABQ18314.1 |
|  | *Cicer arietinum* (Cia) | *S*-adenosyl-l-homocysteine hydrolase | CAI56440.1 |
|  | *Citrus sinensis* (Cs) | Adenosylhomocysteinase-like | XP_006492541.1 |
|  | *Cucumis sativus* (Cus) | *S*-adenosyl-l-homocysteine hydrolase | ADR30776.1 |
|  | *Glycine max* (Gm) | Adenosylhomocysteinase-like | XP_003538528.1 |
|  | *Gossypium hirsutum* (Gh) | *S*-adenosyl-l-homocysteine hydrolase | ACJ11250.1 |
|  | *Medicago truncatula* (Mt) | Adenosylhomocysteinase | XP_003601687.1 |
|  | *Nicotiana tabacum* (Nt) | *S*-adenosyl-l-homocysteine hydrolase | AAV31755.1 |
|  | *Petunia* × *hybrida* (Ph) | *S*-adenosyl-l-homocysteine hydrolase | AAP92452.1 |
|  | *Populus tomentosa* (Pto) | *S*-adenosyl-l-homocysteine hydrolase | KF467171 |
|  | *Populus tomentosa* (Pto) | *S*-adenosyl-l-homocysteine hydrolase | KJ198849 |
|  | *Populus trichocarpa* A (Ptr) | *S*-adenosyl-l-homocysteine hydrolase | XP_002328864.1 |
|  | *Populus trichocarpa* B (Ptr) | Adenosylhomocysteinase family protein | XP_002300147.1 |
|  | *Ricinus communis* (Rc) | Adenosylhomocysteinase | XP_002522805.1 |
|  | *Solanum lycopersicum* (Sl) | Adenosylhomocysteinase-like isoform 1 | XP_004247887.1 |
|  | *Solanum tuberosum* (St) | Adenosylhomocysteinase-like | XP_006349914.1 |
|  | *Theobroma cacao* (Tc) | *S*-adenosyl-l-homocysteine hydrolase | EOY05142.1 |
|  | *Trifolium pratense* (Tp) | Adenosylhomocysteinase | BAE71233.1 |
| Monocotyledons |  |  |  |
|  | *Aegilops tauschii* (Aet) | Adenosylhomocysteinase | EMT01272.1 |
|  | *Setaria italica* (Si) | Adenosylhomocysteinase-like | XP_004953493.1 |
|  | *Hordeum vulgare* (Hv) | *S*-adenosyl-l-homocysteine hydrolase | CAJ01707.1 |
|  | *Oryza sativa* (Os) | Adenosylhomocysteinase-like protein | AAO72664.1 |
|  | *Zea mays* (Zm) | Adenosylhomocysteinase | NP_001148534.1 |
| Prokaryotes |  |  |  |
|  | *Chlorella variabilis* (Cv) | *S*-adenosyl-l-homocysteine hydrolase | EFN51251.1 |
|  | *Dunaliella salina* (Ds) | *S*-adenosyl-l-homocysteine hydrolase | ADJ96635.1 |
