# Supplementary material for: Allelic variation within the S-adenosyl-L-homocysteine hydrolase gene family is associated with wood properties in Chinese white poplar (Populus tomentosa)
Source: BMC Genet. 2014 Jun 20;15(Suppl 1):S4. doi: 10.1186/1471-2156-15-S1-S4 (PMC4118623; doi:10.1186/1471-2156-15-S1-S4)
Supplement: Additional file 1 — Table S1 SAHH protein sequences from species used in this study. Table S2 Primers used for real-time PCR analysis. Table S3 Significant SNP associations (P ≤ 0.05) identified in PtoSAHHA and PtoSAHHB. Table S4 List of significant haplotype-based associations with wood quality and growth traits in the Populus tomentosa association population (n = 460). [file 1471-2156-15-S1-S4-S1.zip › Additional file 1/Table S2.doc]

**Table S2 Primers used for real-time PCR analysis**

| **Gene** | **Forward primer (5′→3′)** | **Reverse primer (5′→3′)** |
| --- | --- | --- |
| *PtoSAHHA* | ATGATCTGCGATCTCGGAGAA | CACCCCCATCCAAAACCTTATTA |
| *PtoSAHHB* | TCCTAGGATTTGTGTTTTGGTGGT | GTGGTCTTCAAGATAGACCGTTT |
| *Actin* | CTCCATCATGAAATGCGATG | TTGGGGCTAGTGCTGAGATT |
