# Supplementary material for: Allelic variation within the S-adenosyl-L-homocysteine hydrolase gene family is associated with wood properties in Chinese white poplar (Populus tomentosa)
Source: BMC Genet. 2014 Jun 20;15(Suppl 1):S4. doi: 10.1186/1471-2156-15-S1-S4 (PMC4118623; doi:10.1186/1471-2156-15-S1-S4)
Supplement: Additional file 1 — Table S1 SAHH protein sequences from species used in this study. Table S2 Primers used for real-time PCR analysis. Table S3 Significant SNP associations (P ≤ 0.05) identified in PtoSAHHA and PtoSAHHB. Table S4 List of significant haplotype-based associations with wood quality and growth traits in the Populus tomentosa association population (n = 460). [file 1471-2156-15-S1-S4-S1.zip › Additional file 1/Table S3.doc]

**Table S3 Significant SNP associations (*P* ≤ 0.05) identified in *PtoSAHHA* and *PtoSAHHB***

| **Trait** | **Locus** | **Position** | **Mutation** | ***P-*value** | ***R*^2^** (%) |
| --- | --- | --- | --- | --- | --- |
| Lignin |  |  |  |  |  |
|  | *PtoSAHHB_435* | Exon 1 | [C : T] | 0.0151 | 3.87 |
|  | *PtoSAHHB_735* | Exon 1 | [G : T] | 0.0307 | 1.13 |
| α-Cellulose |  |  |  |  |  |
|  | *PtoSAHHB_1065* | Intron 1 | [A : G] | 0.0007 | 4.00 |
|  | *PtoSAHHA_1196* | Intron 1 | [C : T] | 0.0191 | 1.29 |
|  | *PtoSAHHA_2203* | 3’-UTR | [G : T] | 0.0064 | 1.76 |
| Holocellulose |  |  |  |  |  |
|  | *PtoSAHHA_1196* | Intron 1 | [C : T] | 0.0056 | 1.80 |
|  | *PtoSAHHA_1028* | Intron 1 | [A : T] | 0.0065 | 1.73 |
|  | *PtoSAHHB_1065* | Intron 1 | [A : G] | 0.0172 | 1.55 |
|  | *PtoSAHHB_618* | Exon 1 | [A : C] | 0.0482 | 1.09 |
| Hemicellulose |  |  |  |  |  |
|  | *PtoSAHHA_51* | 5’-UTR | [G : T] | 0.0471 | 0.92 |
|  | *PtoSAHHA_1028* | Intron 1 | [A : T] | 0.0348 | 1.04 |
|  | *PtoSAHHB_735* | Exon 1 | [G : T] | 0.0188 | 1.33 |
| Fiber length |  |  |  |  |  |
|  | *PtoSAHHB_410* | Exon 1 | [G : A] | 0.0001 | 3.04 |
| Fiber width |  |  |  |  |  |
|  | *PtoSAHHA_1196* | Exon 6 | [T : C] | 0.0139 | 1.42 |
|  | *PtoSAHHA_51* | Exon 6 | [G : T] | 0.0376 | 1.02 |
|  | *PtoSAHHA_1244* | Intron 6 | [G : T] | 0.0385 | 1.01 |
| Microfibril angle (MFA) |  |  |  |  |  |
|  | *PtoSAHHA_1028* | Intron 1 | [A : T] | 0.0413 | 0.97 |
| Diameter at breast height (DBH) |  |  |  |  |  |
|  | *PtoSAHHA_1196* | Intron 1 | [C : T] | 0.0335 | 1.06 |
|  | *PtoSAHHA_1313* | Intron 1 | [A : T] | 0.0381 | 1.01 |
|  | *PtoSAHHA_547* | Exon 1 | [C : T] | 0.0391 | 1.01 |
|  | *PtoSAHHA_1028* | Intron 1 | [A : T] | 0.0432 | 0.96 |
| Tree height (H) |  |  |  |  |  |
|  | *PtoSAHHB_618* | Exon 1 | [A : C] | 0.0004 | 3.46 |
|  | *PtoSAHHA_1313* | Intron 1 | [A : T] | 0.0040 | 1.94 |
|  | *PtoSAHHA_547* | Exon 1 | [C : T] | 0.0164 | 1.35 |
|  | *PtoSAHHA_1627* | Exon 2 | [C : T] | 0.0247 | 1.19 |
| Stem volume (V) |  |  |  |  |  |
|  | *PtoSAHHA_2021* | Exon 2 | [A : T] | 0.0018 | 2.27 |
|  | *PtoSAHHA_1028* | Intron 1 | [A : T] | 0.0165 | 1.35 |
|  | *PtoSAHHA_1313* | Intron 1 | [A : T] | 0.0282 | 1.13 |
|  | *PtoSAHHA_547* | Exon 1 | [C : T] | 0.0430 | 0.96 |

*R*^2^ = percentage of phenotypic variance explained; *Q*-value = correction for multiple tests [FDR (*Q*) ≤ 0.10].
