# Supplementary material for: Allelic variation within the S-adenosyl-L-homocysteine hydrolase gene family is associated with wood properties in Chinese white poplar (Populus tomentosa)
Source: BMC Genet. 2014 Jun 20;15(Suppl 1):S4. doi: 10.1186/1471-2156-15-S1-S4 (PMC4118623; doi:10.1186/1471-2156-15-S1-S4)
Supplement: Additional file 1 — Table S1 SAHH protein sequences from species used in this study. Table S2 Primers used for real-time PCR analysis. Table S3 Significant SNP associations (P ≤ 0.05) identified in PtoSAHHA and PtoSAHHB. Table S4 List of significant haplotype-based associations with wood quality and growth traits in the Populus tomentosa association population (n = 460). [file 1471-2156-15-S1-S4-S1.zip › Additional file 1/Table S4.doc]

**Table S4** **List of significant haplotype-based associations with wood quality and growth traits in the *Populus tomentosa* association population (n = 460).**

| **Trait** | ***P-*value** | ***Q*-value** | ***R*^2^ (%)** | **Significant haplotypes** | **Haplotype frequency** |
| --- | --- | --- | --- | --- | --- |
| α-Cellulose | 0.0017 | 0.0487 | 1.85 | *PtoSAHHA_2203-2222* |  |
|  |  |  |  | G-T | 0.30 |
|  |  |  |  | T-C | 0.33 |
| α-Cellulose | 0.0013 | 0.0399 | 2.17 | *PtoSAHHB_1028-1035-1065* |  |
|  |  |  |  | G-A-G | 0.43 |
|  |  |  |  | T-G-A | 0.40 |
| Holocellulose | 0.0059 | 0.0768 | 2.96 | *PtoSAHHA_1196-1216-1220* |  |
|  |  |  |  | G-G-T | 0.12 |
| Holocellulose | 0.0023 | 0.0579 | 4.01 | *PtoSAHHB_420-435-489-618* |  |
|  |  |  |  | C-T-C-A | 0.47 |
|  |  |  |  | A-C-T-C | 0.35 |
| Hemicellulose | 0.0051 | 0.0768 | 2.50 | *PtoSAHHB_718-735* |  |
|  |  |  |  | G-G | 0.41 |
|  |  |  |  | C-T | 0.43 |
| Fiber width | 0.0022 | 0.0579 | 3.2 | *PtoSAHHA_1196-1216-1220-1244* |  |
|  |  |  |  | G-G-T-T | 0.26 |
|  |  |  |  | T-T-G-G | 0.18 |
| D | 0.0006 | 0.0192 | 2.70 | *PtoSAHHA_826-1003-1028* |  |
|  |  |  |  | G-T-A | 0.18 |
|  |  |  |  | A-G-T | 0.41 |
| H | 0.0015 | 0.0487 | 1.86 | *PtoSAHHA_517-547-568* |  |
|  |  |  |  | T-T-T | 0.15 |

DBH *=* Diameter at breast height; V = stem volume; *R*^2^ = percentage of phenotypic variance explained; *P-*value = significant level for haplotype-based association (significance is *P* ≤ 0.05); *Q*-value = a correction for multiple tests [FDR (Q) ≤ 0.10];
